# Secret Remedies and Branded Products—VII

**Published:** 1908-07-04

**Authors:** 


### SECRET REMEDIES AND BRANDED PRODUCTS—VII.

If a proprietary medicinal compound represents an advance in pharmaceutical science there is some excuse for its existence, and a manufacturer who introduces a valuable new product justly deserves protection. Whether he ought to have the unlimited protection afforded by trade-marks is open to serious question, but upon one point every thinking person will be agreed—namely, that the originator of a valuable substance is entitled to infinitely more protection than the mere coiner of a fancy name. The man who discovers some new manufacturing process which is, from the medical standpoint, of any real value has just as much right as any other inventor to the protection afforded by the Patent Acts. He discloses his process, takes out Letters Patent, and is granted by the State an absolute monopoly in the working of his invention for a term of fourteen years. On no ground could his conduct be assailed as improper. There is insured to him an adequate opportunity for profiting by the use of his brains, and at the same time the taint of secrecy, so fatal from the point of view of the conscientious prescriber, does not attach to his product. Moreover, if for any improper reason he materially alters his process, he must either declare the alteration or run the risk of losing his monopoly. It is unfortunate for the reputation of the genuine inventor and for the public at large that, in regard to medicinal articles and processes, it is usually open to him to secure a quasi-monopoly—not for fourteen years only, but for an unlimited time—by availing himself of the "Trade-mark" method, instead of the "Patent" method, of securing proprietary rights. For the purpose of the trade-mark laws there need be nothing of the slightest value in the invention—there need be no invention at all—and no disclosure of the nature of the article sold under the trade-mark need be made. This would be a serious matter, even if all medicinal articles sold under trade-marks were valuable or even innocuous.

In the vast majority of cases the object the manufacturer has in view in christening his articles with fancy names is to create a monopoly, and so place himself in a position to shirk competition and charge fancy prices. Unfortunately the law plays into the hands of such people, and not only protects them from competition, but actually places them outside the law relating to adulteration. Take, for instance, the case of "Compound Rhubarb Pill"; this is composed of rhubarb root, Socotrine aloes, and other ingredients, and if a pharmacist dispenses a pill which is not in accordance with the formula of the British Pharmacopœia when this pill is prescribed, he commits an offence for which he renders himself liable to a heavy penalty. On the other hand, it is open to any manufacturer to make pills according to the official formula and offer them under some such title as "Pilode Rhei et Aloes Co."; but supposing Socotrine aloes to be very dear and Barbadoes aloes to be very cheap, there is nothing to prevent him from changing his original formula and substituting the cheaper variety for the more costly. In making a charge for twelve compound rhubarb pills the pharmacist takes into account the cost of the ingredients, the time and skill required to compound them, and the fact that there are something like ten thousand other pharmacists who are able to supply them. On the other hand, the ingenious proprietor of the "Pilode" brand, in fixing his price, is not hampered by any considerations of what anyone else is charging, but the chief question to consider is how much he can get for them.

Again, it may occur to a manufacturer that liniment of camphor is a useful substance to exploit under a fancy name. When camphor is 1s. 10d. per lb and olive oil is at a normal figure, he will probably be satisfied with the Pharmacopœia formula, and a price that is four times that of the ordinary trader; but when camphor advances to 4s. 8d., as it recently did, the law is powerless to prevent him from leaving out half the camphor and substituting nut oil for olive oil, although for this very offence large numbers of small traders have been heavily penalised during the time camphor has been so costly. To illustrate another way in which the use of a fancy name may enable the manufacturer of a secret compound to evade his obligations one may instance an article containing some such substance as acetanilide, which at some future date may be scheduled as a poison under the Pharmacy Acts. If such a time arrives, makers of all compounds containing acetanilide must either label their product "poison" or substitute some other substance; but nobody can compel them to notify practitioners who are accustomed to prescribe this particular compound that a change in composition has been made. This, in fact, has been the case in America, where the manufacturers of a compound which is very largely prescribed in this country, have, as a result of the enactment of a law requiring that all compounds containing acetanilide shall be labelled to that effect, substituted phenacetin for acetanilide. It will be seen that the owner of a secret compound, by merely claiming secrecy, gains an advantage over the maker of an article whose composition is declared; for the selling price of a secret compound is not regulated by the ordinary rules of healthy commerce, and the maker of it is not subservient to a law enacted for the purpose of protecting the public from fraud.

It is admitted that there are many excellent proprietary products on the market, and the owners of these products have absolutely nothing to fear from any exposure of fraudulent nostrums. On the other hand, there are black sheep in the proprietary-medicine trade, just as there are in every other. Are we, then, wise in placing implicit confidence in a class which must comprise some who will take unfair advantage of that confidence? When a physician prescribes a compound for which a standard has been laid down, he knows that a pharmacist who supplies something that is not prepared exactly in accordance with the standard formula commits an offence for which he may be punished. But if the same physician prescribes a proprietary product, for which there is no standard, his patient may be supplied with one article one day and another the next, and the maker of the compound entails no risk.

